# When Ascites Hides More: A Diagnostic Dilemma in an Unusual Presentation

**DOI:** 10.7759/cureus.76721

**Published:** 2025-01-01

**Authors:** Abhinav Gupta, Varun Mehta, Manjeet K Goyal, Manisha Khubber, Yogesh K Gupta

**Affiliations:** 1 Gastroenterology, Dayanand Medical College and Hospital, Ludhiana, IND; 2 Gastroenterology and Hepatology, Dayanand Medical College and Hospital, Ludhiana, IND

**Keywords:** diagnostic challenges, eosinophil counts, eosinophilic ascites, eosinophilic gastro-intestinal disorders, eosinophilic gastrointestinal disorders, infectious and parasitic diseases, steroid use

## Abstract

Eosinophilic ascites, a rare manifestation of eosinophilic gastroenteritis, is characterized by eosinophilic infiltration in the gastrointestinal tract and ascitic fluid. In this case, a 60-year-old male presented with abdominal pain and vomiting, with diagnostic challenges due to non-specific symptoms. The diagnosis was made based on history and eosinophil-rich ascites. Treatment with corticosteroids resulted in symptom improvement. This case emphasizes the importance of early recognition and prompt treatment to avoid unnecessary surgical intervention in patients with unexplained ascites.

## Introduction

Eosinophilic gastrointestinal diseases (EGIDs) are chronic, immune-mediated disorders of the gastrointestinal tract that are characterized by abnormal eosinophil-predominant tissue inflammation. These diseases can occur at any age but are usually present in the third to fifth decades of life [1,2]. The pathogenesis remains unclear, although multiple studies postulate possible allergic components as 50-70% of patients have concomitant atopic diseases [1,3,4]. The clinical presentation varies based on the location and extent of bowel involvement.

EGIDs are classified into mucosal, muscular, and serosal types [5,6]. Mucosal disease (70%), the most common form, causes non-specific symptoms, while muscular involvement (20%) leads to motility issues and obstruction [7-9]. Serosal disease (10%), the rarest, presents with eosinophilic ascites in isolation or along with symptoms of mucosal and/or muscular disease [1]. This case report presents a rare occurrence of eosinophilic ascites in a patient presenting with abdominal pain, highlighting diagnostic challenges, therapeutic interventions, and clinical outcomes.

## Case presentation

**Figure 1:**
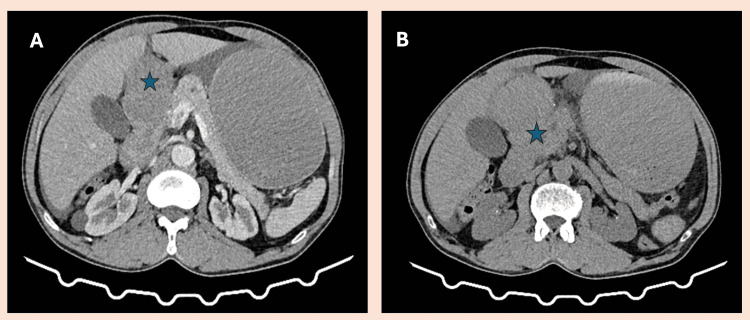
Contrast-enhanced CT scan illustrating duodenal wall thickening (A) and (B) axial contrast-enhanced CT image shows circumferential wall thickening in the duodenum (indicated by the star).

**Figure 2:**
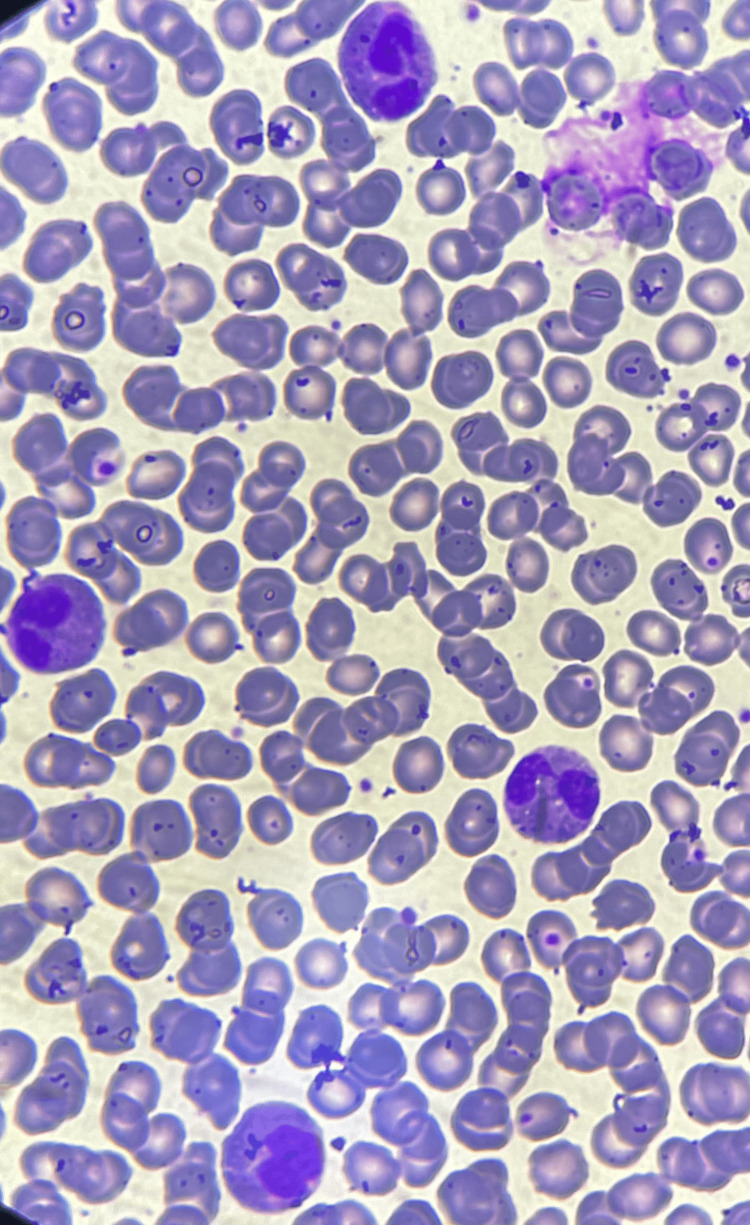
Peripheral blood smear showing eosinophilia (Wright-Giemsa stain, 1,000× magnification). Peripheral blood smear revealing marked eosinophilia with abundant bi-lobed eosinophils.

**Figure 3:**
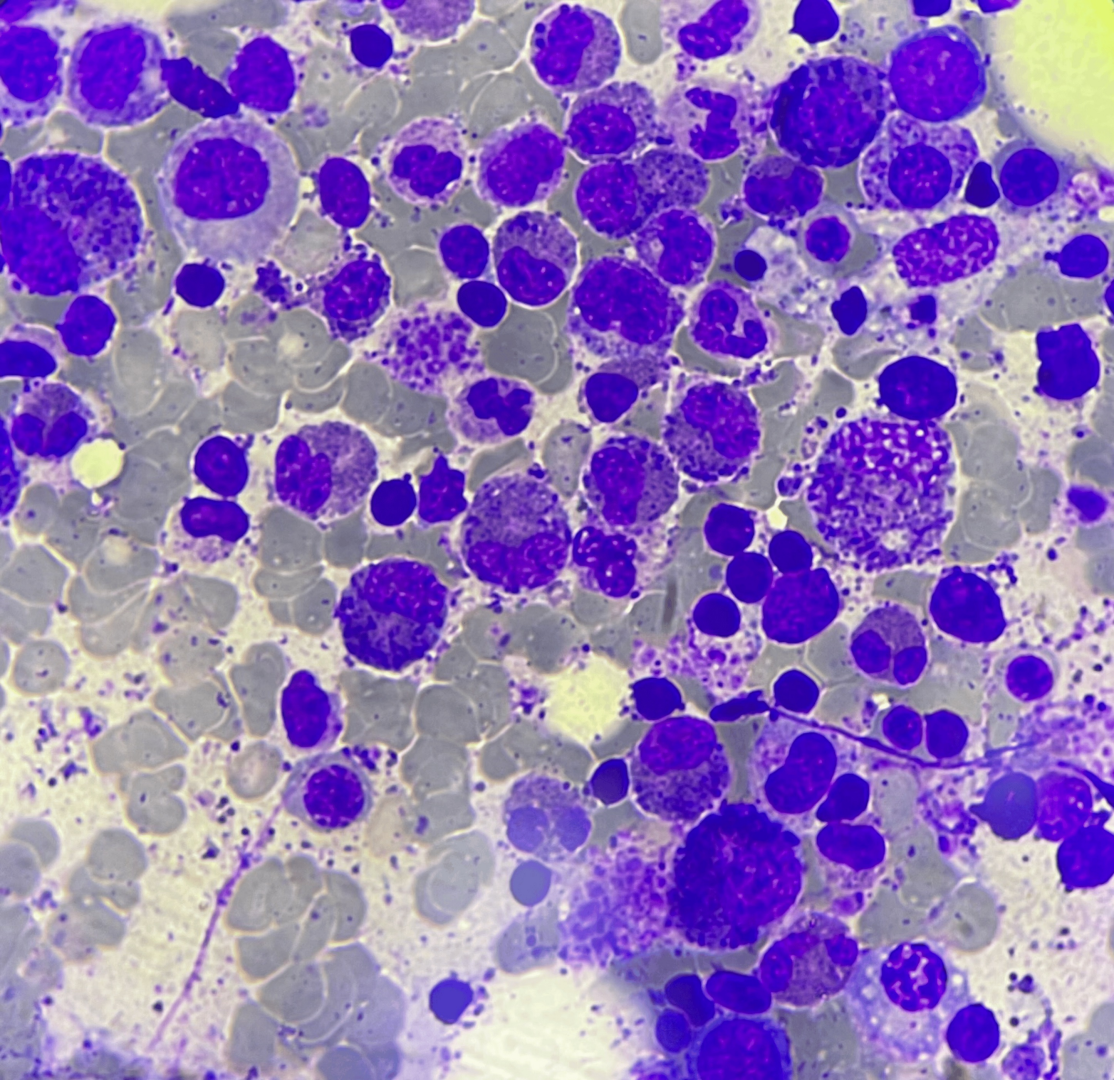
Bone marrow aspirate illustrating marked eosinophilic hyperplasia with increased eosinophilic precursors (Wright-Giemsa stain, 1,000× magnification).

**Figure 4:**
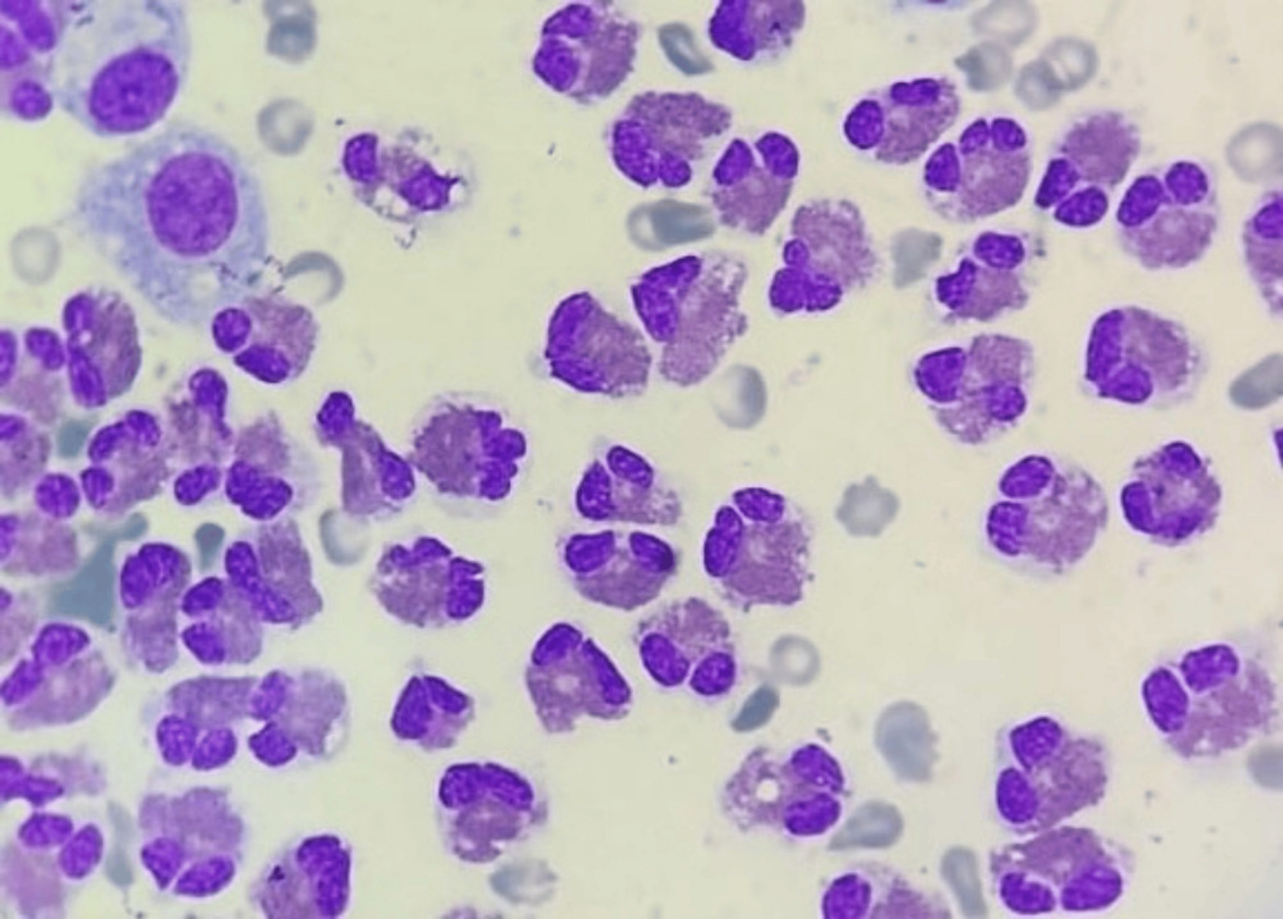
Ascitic fluid cytology with an eosinophilic predominance (Wright-Giemsa stain, 400× magnification)

A 60-year-old male presented with abdominal pain and vomiting for one week. This was his first presentation and his first-ever hospitalization. There was no personal or family history of asthma, allergic rhinitis, atopic dermatitis, seasonal allergies, or any other allergic disorder. Clinical examination was unremarkable. His routine laboratory tests revealed normal hemoglobin (139 g/L), WBC count (11.8 × 10^9^/L) (Table 1), and normal platelet count (265 × 10^9^/L). The comprehensive metabolic profile was within normal limits. Urinalysis, ECG, and chest X-ray examination were also normal. Ultrasonography of the abdomen was suggestive of minimal free fluid in the abdomen, which was not amenable to aspiration. He was treated empirically with anti-spasmodic and anti-emetics. However, his symptoms persisted, and therefore, a contrast-enhanced computed tomography of the abdomen and pelvis was done, which revealed circumferential wall thickening involving the stomach and the first part of the duodenum (Figure 1). He underwent an upper gastrointestinal endoscopy, which was normal, and segmental biopsies were taken for histopathological analysis. IgG4 levels and tumor markers were normal. A diagnostic laparoscopy was planned. The subsequent day hemogram revealed a total leukocyte count (TLC) of 11.3 × 10^9^/L with eosinophil predominance (49.6%) and an absolute eosinophil count of 5.6 × 10^9^/L (Figure 2). This prompted a work-up of eosinophilia. His bone marrow aspiration (Figure 3) and biopsy were suggestive of normocellular marrow with a prominence of eosinophils and eosinophil precursors (20% eosinophils, 22% myelocytes, and 16% metamyelocytes). His antineutrophil cytoplasmic antibodies (ANCA) and antinuclear antibody (ANA) profile were negative (Table 2). His 2D echocardiogram was also normal. Moreover, during the course of hospitalization, he developed abdominal distension. Abdominal paracentesis was done, which revealed a low serum ascites albumin gradient (SAAG) (1.07), with a cell count of 4.48 × 10^9^/L with 90% eosinophils (4.03 × 10^9^/L, Table 3, Figure 4). Esophageal, gastric, antral, and duodenal biopsies revealed non-specific inflammation with normal eosinophil count. He was given the option of laparoscopic full-thickness biopsy and resection in view of eosinophilic ascites, but he did not consent to the same. In view of the lack of an alternate explanation and as per the diagnostic criteria suggested by Talley et al. (Table 4), he was started on oral steroids.1 He responded to the treatment and symptomatically improved. His repeat hemogram (fifth day of stating steroid) revealed an absolute eosinophil count of 5.5 × 10^9^/L. He was subsequently discharged and followed up on an outpatient basis. On serial follow-up, the patient was completely free of symptoms and maintained his health even after steroids were gradually tapered and stopped, and his eosinophil count turned out to be normal. He is currently under follow-up for more than a year.

**Table 1:** Laboratory investigation of the patient AEC - absolute eosinophil count, ALP - alkaline phosphatase, ALT - alanine aminotransferase, AST - aspartate aminotransferase, NAD - nothing abnormal detected, PA - posteroanterior, WBC - white blood cell

Data	Day 1	Day 4	Day 7	Day 21
Hemoglobin (12-15 g/L)	139	131	130	133
WBC count (4-10 × 10^9^/L)	11.8	11.3	13.9	8.2
Neutrophils (40-80%)	49.4	26.8	36.7	49.3
Lymphocytes (20-40%)	23.7	18.4	18.7	37
Monocytes (2-10%)	5.2	4	4.2	7
Eosinophils (1-6%)	20.1	49.6	39.9	5.9
Basophils (0-1%)	1.6	1.2	0.5	0.8
Absolute eosinophil count (0.1-0.3 × 10^9^/L)	2.38	5.6	5.5	-
Platelet count (150-450 × 10^9^/L)	265	261	412	250
Serum bilirubin (1.71-20.52 µmol/L)	4.78	-	-	-
AST (0-40 U/L)	20	-	-	-
ALT (0-41 U/L)	10	-	-	-
ALP (40-129 U/L)	76	-	-	-
Total protein (66-87 g/L)	56	-	-	-
Serum albumin (35-52 g/L)	35	-	-	-
Serum urea (7.14-35.71 mmol/L)	12.49	-	-	-
Serum creatinine (44.21-97.26 µmol/L)	86.63	-	-	-
Serum sodium (136-148 mmoL/L)	140	-	-	-
Serum potassium (3.5-5 mmoL/L)	4.21	-	-	-
Random blood sugar (3.885-7.77 mmol/L)	5.11	-	-	-
Urine routine	NAD	-	-	-
Chest X-ray (PA view)	NAD	-	-	-
Stool routine	Normal	-	-	-
Ultrasound abdomen	Minimal ascites	-	Mild to moderate ascites	-

**Table 2:** Work-up for peripheral eosinophilia to rule out other causes ANA - antinuclear antibody, ANCA - antineutrophil cytoplasmic antibodies, ELISA - enzyme-linked immunosorbent assay, HIV - human immunodeficiency virus, IF - immunofluorescence, Ig - immunoglobulin (with class), PBF - peripheral blood film

Investigations	Result
PBF for immature/dysplastic cells	Negative
Vitamin B12 (160-950 pg/mL)	201
IgE, IgM, IgG	Normal
HIV - 1 and 2 (ELISA)	Negative
Echocardiography	Normal
Cardiac enzymes	Normal
Pulmonary function tests	Normal
Stool multiplex array	Negative
Bone marrow aspiration and biopsy	Normal
ANA (IF)	Negative
ANA profile	Negative
ANCA profile	Negative

**Table 3:** Ascitic fluid analysis for this patient ADA - adenosine deaminase, AFB - acid-fast bacillus

Parameter	Value
Albumin (3.1-17.7 g/L)	23.4
Protein (<25 g/L)	38
Glucose (3.885-5.55 mmol/L)	4.77
Total cells (<0.3 × 10^9^/L)	9.85
Eosinophil count (0.003-0.009 × 10^9^/L)	9.3
ADA (<30 U/L)	20
Gram stain	Negative
Culture	No growth
AFB stain	Absent
Mycobacterial culture	No growth
Fungal culture	No growth

**Table 4:** Diagnostic criteria for eosinophilic ascites related to eosinophilic gastrointestinal disorders and the presentation in this case meeting the criteria [1]

Feature	Checklist in our case
Gastrointestinal symptoms	Vomiting and pain abdomen ascites
Tissue diagnosis	Eosinophil-rich ascites
No extraintestinal involvement/alternate explanation	Yes

## Discussion

EGIDs are increasingly recognized worldwide, yet eosinophilic ascites, a rare manifestation of this condition, present a diagnostic challenge due to their non-specific clinical features and overlap with more common causes of ascites [7]. Eosinophilic serositis is a rare condition characterized by inflammation of serosal membranes accompanied by an eosinophilic infiltrate. It is a part of the spectrum of EGIDs, with multifactorial pathogenesis, with proposed mechanisms including hypersensitivity reactions, parasitic infections, autoimmune processes, and idiopathic causes [8].

The differential diagnosis of eosinophilic ascites includes infections, malignancies, inflammatory bowel disease, hypereosinophilic syndrome, and vasculitides [10]. Diagnosis requires a detailed history to exclude drug-induced eosinophilia, ingestion of raw or uncooked meat, and dietary sources of parasites (Table 5). A comprehensive personal history, including residence, sanitation, and travel to endemic areas, is vital. Physical examination aids in detecting other causes of eosinophilia and extraintestinal involvement. Furthermore, lymphoproliferative disorders such as Hodgkin lymphoma and certain peripheral T-cell lymphomas derived from CD4 cells, including Sézary syndrome, adult T-cell leukemia/lymphoma, and angioimmunoblastic T-cell lymphoma, also need to be carefully ruled out as these, although rare, can also lead to eosinophilia Laboratory tests, stool studies, ascitic fluid analysis (as in this case), and endoscopy is of utmost importance. While negative mucosal biopsies do not exclude muscular or serosal disease, a laparoscopic full-thickness biopsy or endoscopic mucosal resection (EMR) may help. Diagnosis hinges on eosinophil-rich ascitic fluid and peripheral eosinophilia, necessitating high clinical suspicion [11].

**Table 5:** Classification of eosinophilic gastrointestinal disorders along with presentation and prevalence as described by Klein et al. [5]

Sub-type	Presentation	Estimated prevalence
Mucosal	Non-specific (depends on area involved) abdominal pain nausea vomiting early satiety diarrhea weight loss malabsorption protein-losing enteropathy failure to thrive	≈70%
Muscular	Wall thickening and impaired motility gastric outlet obstruction Intestinal obstruction Pseudoachalasia or esophageal stricture dysphagia regurgitation perforation	≈20%
Serosal	Isolated ascites; ascites with above features pleural effusion	≈10%

Eosinophils, typically present in the lamina propria, play an important role in gastrointestinal mucosal immunity. Their numbers increase during inflammatory processes such as parasitic infections and allergic diseases. Activated eosinophils release inflammatory mediators like eosinophil cationic protein and eosinophil-derived neurotoxin, which are cytotoxic to the gastrointestinal epithelium. This triggers mast cell degranulation and release of cytokines (e.g., IL-4, IL-5), chemokines (e.g., eotaxin), and lipid mediators. Th2-type immune responses, particularly elevated cytokines and eotaxin, are critical in eosinophilic gastroenteritis, and with serosal involvement, there is the formation of inflammatory exudative fluid rich in eosinophils [11]. 

EGIDs generally respond well to corticosteroids, the primary treatment for inducing remission, with an 80% efficacy rate. Symptoms typically improve within one week, and eosinophil levels normalize within two weeks [12]. Alternative treatments, such as antihistamines, montelukast, and sodium cromoglycate, may be useful in cases of relapse, which occurs in about 50% of patients post-steroid discontinuation [13]. Immunosuppressive agents like azathioprine or cyclophosphamide may be considered for steroid-resistant cases [14]. Dietary interventions, such as six-food or elemental elimination diets over four to six weeks, may induce remission in some patients [15]. Emerging therapies include anti-Siglec-8 antibody (Lirentelimab), anti-cytokine (suplatast tosilate), anti-integrin (vedolizumab), and anti-IL5 antibody (benralizumab) [16-19].

While EGIDs are being increasingly diagnosed based on a high index of suspicion among gastroenterologists these days, our case was unusual due to the presence of stomach and duodenal mass, which may have contributed to his symptoms. Taking this into consideration, along with the fact that ours is a country with a high incidence of helminth infection, this could very well have been passed under the knife and might have imposed additional morbidity on the patient. The point of discrimination was the worsening of ascites along with the increase in peripheral eosinophilia, which led to further evaluation in this patient, something which could have easily not turned up. This case adds to the limited body of literature on eosinophilic ascites and underscores the need for clinicians to consider this diagnosis in patients with unexplained ascites, particularly when eosinophilia is present.

## Conclusions

Early diagnosis of EGIDs is essential to prevent unnecessary surgeries and allow timely management. Recognizing clinical signs like peripheral eosinophilia, ascites, and steroid responsiveness is critical, especially in parasitic-endemic regions. This case highlights key diagnostic indicators and the effectiveness of corticosteroids in managing EGIDs.
